# Electronic properties of the Sn_1−x_Pb_x_O alloy and band alignment of the SnO/PbO system: a DFT study

**DOI:** 10.1038/s41598-020-73703-y

**Published:** 2020-10-08

**Authors:** N. Kelaidis, S. Bousiadi, M. Zervos, A. Chroneos, N. N. Lathiotakis

**Affiliations:** 1 Theoretical and Physical Chemistry Institute, National Hellenic Research Foundation, Vass. Constantinou 48, 11635 Athens, Greece; 2 Faculty of Engineering, Environment and Computing, Coventry University, Priory Street, Coventry, CV1 5FB UK; 3 Faculty of Physics, National and Kapoditrian University of Athens, Panepistimiopolis, Zografos, 157 84 Athens, Greece; 4 Nanostructured Materials and Devices Laboratory, School of Engineering, University of Cyprus, PO Box 20537, 1678 Nicosia, Cyprus; 5 Department of Materials, Imperial College, London, SW7 2AZ UK

**Keywords:** Materials science, Materials for devices, Materials for energy and catalysis, Materials for optics

## Abstract

Tin monoxide (SnO) has attracted attention due to its p-type character and capability of ambipolar conductivity when properly doped, properties that are beneficial for the realization of complementary oxide thin film transistors technology, transparent flexible circuits and optoelectronic applications in general. However, its small fundamental band gap (0.7 eV) limits its applications as a solar energy material, therefore tuning its electronic properties is necessary for optimal performance. In this work, we use density functional theory (DFT) calculations to examine the electronic properties of the Sn_1−x_Pb_x_O ternary oxide system. Alloying with Pb by element substitution increases the band gap of SnO without inducing defect states in the band gap retaining the anti-bonding character of the valence band maximum which is beneficial for p-type conductivity. We also examine the properties of the SnO/PbO heterojunction system in terms of band alignment and the effect of the most common intrinsic defects. A broken gap band alignment for the SnO/PbO heterojunction is calculated, which can be attractive for energy conversion in solar cells, photocatalysis and hydrogen generation.

## Introduction

Tin monoxide (SnO), or stannous oxide, is a layered semiconductor oxide that has attracted significant attention mainly due to its p-type character, first reported in 2001^1^ and due to the capability of bipolar conductivity through doping^2^. Therefore, like its counterpart tin dioxide (SnO_2_), SnO is studied as a promising material for electronic and optoelectronic applications such as displays, transparent electrodes for photovoltaics, gas sensors and lithium batteries. The possibility of a p-type oxide with relatively high hole mobility and charge density can have various technological applications, as for example on the technology of thin film transistors (TFTs) by providing the p-type counterpart of a complementary metal oxide semiconductor (CMOS) inverter^3^. In photovoltaics (PV), they can be used as electrodes or interconnect layers that enable carrier collection or even lead to an all-oxide PV as next generation solar cells^4^. The electronic properties of SnO, such as highly dispersive bands and low hole effective mass, are very promising for high efficient oxide photovoltaics without the stability issues of perovskites. However, its efficiency as a solar energy material is limited by its small fundamental band gap^2,4^.

The inherent p-type character of SnO has been attributed to unintentional Sn-vacancies^5,6^. It has also been reported that hydrogen, which can be present as an unintentional impurity in most growth environments, forms complexes with Sn vacancies facilitating Sn vacancy formation^7^. It has been suggested^7^ that H impurities and Sn vacancies act as shallow acceptors, therefore contributing to a p-type conductivity.

Doping of SnO can modify its conductivity. A number of dopants have been examined in the literature such as Ag^8^, Cl^9^, Sb^10^ but some studies have shown contradictory results. For example, the substitutional doping of Sb lead to enhanced hole concentrations in some cases^11^ or to decreased hole mobility and concentration in others^4^. Obtaining n-type conductivity in SnO is considered difficult due to the low formation energy of the Sn + 2 vacancy, so the realization of the bipolar nature is challenging^12^.

Growth of SnO has been performed by various methods such as atomic layer deposition (ALD)^13^, DC magneton sputtering^14^, pulsed layer deposition (PLD)^15,16^, chemical spray pyrolysis^17^, exfoliation^18^ and thermal decomposition^19^. Accurate control of the oxidation state of Sn is challenging and often SnO_2_ and other phases are produced. However, this is not always a disadvantage, e.g. Caraveo-Frescas et al.^14^ showed that residual tin increased mobility. In most cases, the growth of SnO will also yield non stoichiometric compound as in the studies of Refs.^13–19^.

Besides all the above efforts, the structural and electronic properties of SnO have also been studied in the past using density functional theory (DFT). Recently, an extensive study for the most promising n- and p- type dopants has been published^20^ where Li, Na, K, Cs, Rb and Ag were were found to act as shallow acceptors and therefore were proposed for p-type doping of SnO at a substitutional position, replacing Sn. Additionally, isovalent Sn_1−x_M_x_O (M = Mg, Ca, Sr, Zn) alloys have been examined theoretically for the application of SnO as a thin film based solar cell^2^. It was found that the optical and direct band gap can be tailored without the presence of defect states in the gap and SnO-based alloys can be promising for energy conversion such as photovoltaics.

Another element that is isovalent to Sn but with a larger atomic radius is Pb. Pb has already been investigated as a dopant for SnO_2._ Initially, it was reported by Lappe^21^ that the alloying of Pb in Sn substitutional positions results in a shift of the absorption spectrum of SnO_2_ to lower energies, increasing at the same time its conductivity. It was also found that the lattice constant of Pb_x_Sn_1−x_O_2_ layers increased linearly with x. On a recent theoretical work by Butler et al.^22^, the heteroepitaxy of ultrathin oxide layers, such as PbO_2_, on SnO_2_ was proposed as a method to fine tune its electronic properties e.g. work function for various applications such as gas sensing^23^.

Contrary to efforts on Pb doping of SnO_2_, Pb_x_Sn_1−x_O_2_ and the PbO_2_/SnO_2_ heterojunction (HJ), there are very few investigations on Pb doping of SnO and the properties of the ternary oxide Pb_x_Sn_1−x_O. Pb doping of SnO was investigated experimentally by Liao et al.^16^ who observed a small increase of the optical band gap by 0.7 eV (to 2.75 eV) and a decrease of the hole mobility, attributed possibly to trap formation. The preparation of SnO/PbO solid solution and its properties were examined by Kwestroo et al.^24^, whereas Lim et al.^20^ investigated the properties of layered SnO and layered PbO for energy applications. They concluded that SnO is a better material for electron transfer and a better catalyst for hydrogen evolution than PbO. PbO and SnO have also been used separately as anodes in Li batteries^25,26^, therefore they are also attractive for energy storage. PbO has an energy band gap of 2.59 eV^27^ and an electron affinity χ = 0.7 eV. Additionally, PbO can be grown containing O vacancies (Pb rich PbO) showing n-type conductivity or PbO can contain O interstitials (O rich PbO) becoming p-type^28^. Conversely, SnO is a p-type semiconductor with an energy band gap of 0.7 eV and a larger electron affinity of χ = 3.7 eV^15,29^. Consequently, it should be possible to tune the properties e.g. energy band gap, work fucntion and electron affinity of the ternary oxide Pb_x_Sn_1−x_O which can be important for the development of electronic and optoelectronic devices. In addition, the deposition of p-type SnO over n-type is interesting as it could lead to a p-n heterojunction, that may be attractive for energy conversion and storage applications.

Herein, we perform DFT calculations and we study the structural and electronic properties of Pb-doped SnO with moderate to high Pb content resulting in the ternary oxide Pb_x_Sn_1−x_O*.* We also analyze the orbital contributions in the states of the valence and conduction bands and then examine the properties of the SnO/PbO heterojunction such as its band alignment that is important for heterostructure in energy conversion applications. The effect of intrinsic defects on the band alignment is also investigated and more specifically we consider the case of Sn vacancies in SnO and O vacancies in PbO.

## Results and discussion

### Structural and electronic properties of undoped SnO

**Figure 1:**
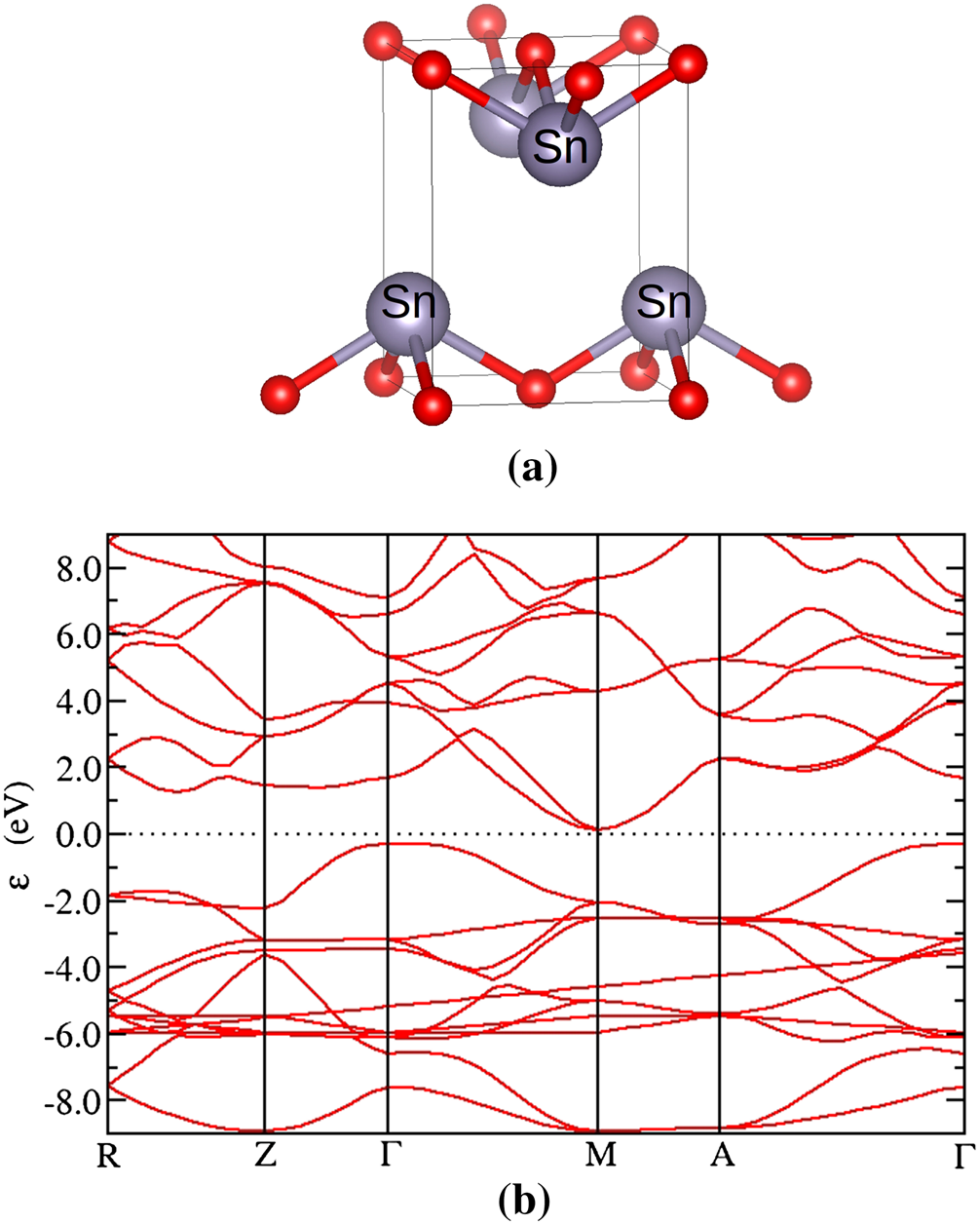
(**a**) SnO litharge structure (**b**) band structure.

The most stable form of SnO is the litharge, i.e. the tetragonal structure of a-PbO, shown in Fig. 1a. Its space group is P4/nmm with lattice parameters of a = 3.801 Å, c = 4.835 Å^30^. Our calculated lattice parameters are shown in Table 1 and are in agreement with previous theoretical work^31^. When including the semi-empirical long-range dispersion correction by Grimme^32^ to account for the Van der Waals interactions, as implemented by the CASTEP code^31^, the lattice c which is associated with distance between layers is calculated at 4.842 Å, very close to the experimental value^36^.

**Table 1:** Calculated structural properties.

a (Å)	c (Å)	Volume of unit-cell (Å^3^)	Exchange correlation functionals
3.863	4.997	74.780	PBE—GGA
3.830	4.891	71.745	PBE—VdW *Tkatchenko—Scheffler*
3.839	4.829	71.052	PBE-VdW *Grimme*
3.80	4.83	69.745	experiment

Regarding the electronic properties, it is known that SnO has a small indirect gap of 0.7 eV found between the Γ-point of the valence band maximum (VBM) and the M-point of the conduction band minimum (CBM)^33^. The direct band gap at the M-point is 2.7 eV. The perfect litharge cell used for calculations is shown in Fig. 1a along with the calculated band structure in Fig. 1b. The indirect band gap calculated with LDA formalism is 0.4 eV between G and K points and the direct band gap at the M point is calculated at 2 eV. The underestimation of the band gaps (optical and fundamental) is expected in DFT calculations. We found the CBM at the M-point of the Brillouin zone and the VBM at the Γ-point, in agreement with previous studies.

In the literature, the calculated band gap of SnO has been found to depend to a great extend on the lattice parameters and the adoption of the PBE relaxed structure was deemed necessary to obtain a non-zero band gap with DFT^34^. In accordance, we find that the electronic properties are better described by the PZ^35^ local exchange–correlation functional (LDA). The band gap with this method is calculated at ~ 0.4 eV.

**Figure 2:**
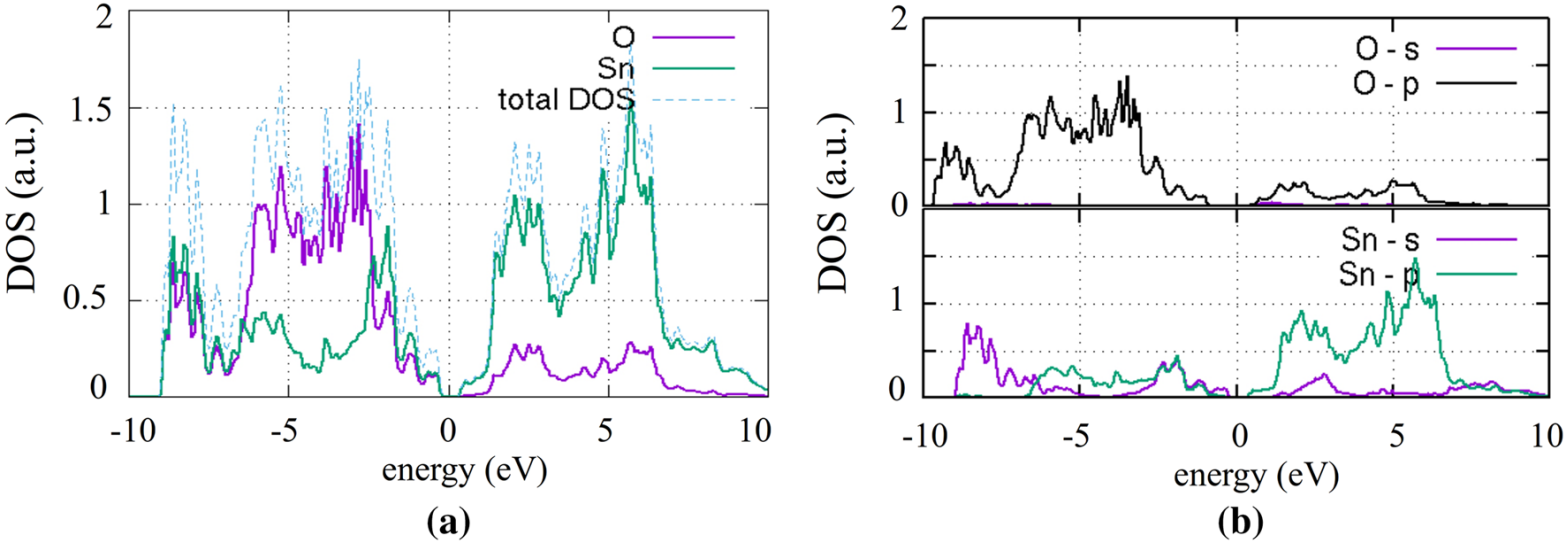
(**a**) Partial (ion projected) DOS and (**b**) partial (orbital projected) DOS of perfect SnO.

The total (a) and partial (b) density of states (DOS) are shown in Fig. 2. The first part of the valence band, at the region between – 10 and − 7 eV is formed by the O-p and Sn-s orbitals. Near the VBM, the Sn–p states also contribute in the mixing: the O-p, Sn-s and Sn-p orbitals dominate the DOS at the region of − 7 eV to the VBM. At the onset of the conduction band, the main contribution originates mostly from the Sn-p and the O-p orbitals (Fig. 2b) with a contribution of the Sn-s around 2 eV. The presence of the cation (Sn) s—states at the VBM is significant and the Sn 5s^2^ electrons interact with the anion (O) p—states of the valence band (Fig. 2b), creating bonding (bottom of the valence band) and anti-bonding states (top of upper valence band), giving rise to the lone pair formation, according to the analysis presented by Welsh et al.^36^. The strong presence of the Sn p-states aids in the stabilization of the anti-bonding states.

### Ιmpact of Pb content on the structural and electronic properties of Sn_1−x_Pb_x_O

To calculate the effect of Pb doping, DFT calculations were performed on a 3 × 3 × 3 108-atom supercell. For higher Pb-concentrations a 2 × 2 × 2 supercell was used, which consists of a total of 32 atoms. As in the undoped case, the PBE functional was applied for the geometry optimization. Pb doping was increased to alloying levels and concentrations of up to 50% were examined. For each alloying level, various configurations were examined and the ones with the minimum formation energy were selected as the most probable. Furthermore, the electronic properties of undoped litharge PbO (α-PbO) and Sn-doped PbO were examined, as a way to examine the other end of the Pb-doping spectrum as both structures are litharge type.

**Figure 3:**
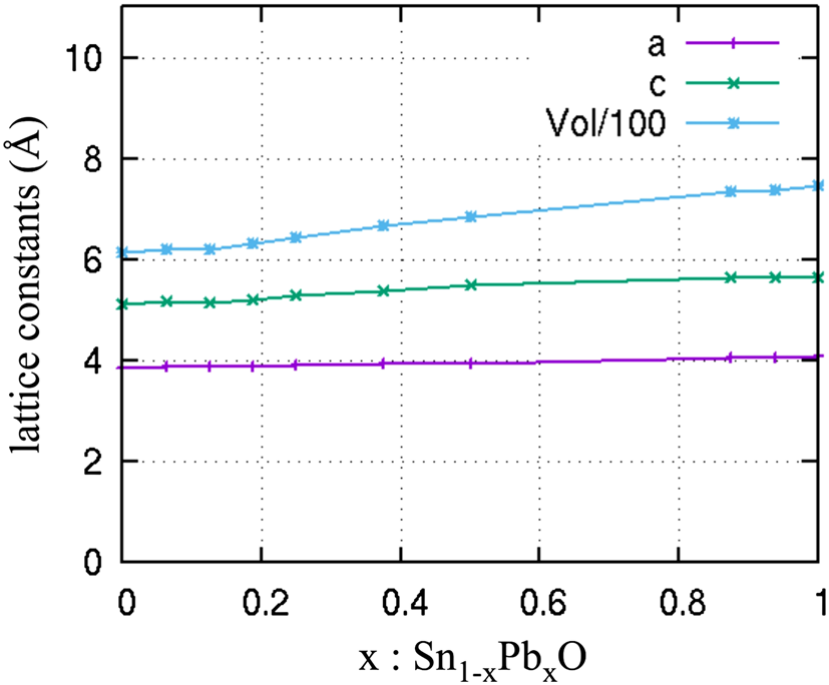
Lattice constants a, c (in Å) and Volume (in Å^3^, divided by 100) of Sn_1−x_Pb_x_O vs x.

The lattice constants of the calculated structure and its cell volume versus the Pb concentration are plotted in Fig. 3. As expected due to its size, Pb increases monotonically the lattice parameters. This represents the ideal case where Pb is dissolved in the tetragonal SnO phase. This is not always the case in experiment. In an early work by Kwestroo et al.^24^, single-phase solid solutions of Pb_1−x_Sn_x_O were obtained at x < 0.25, whereas in the work by Liao et al., doping with Pb concentration up to 0.30 did not change the lattice parameters, indicating that Pb was not dissolved in the tetragonal phase during this experiment^16^.

**Figure 4:**
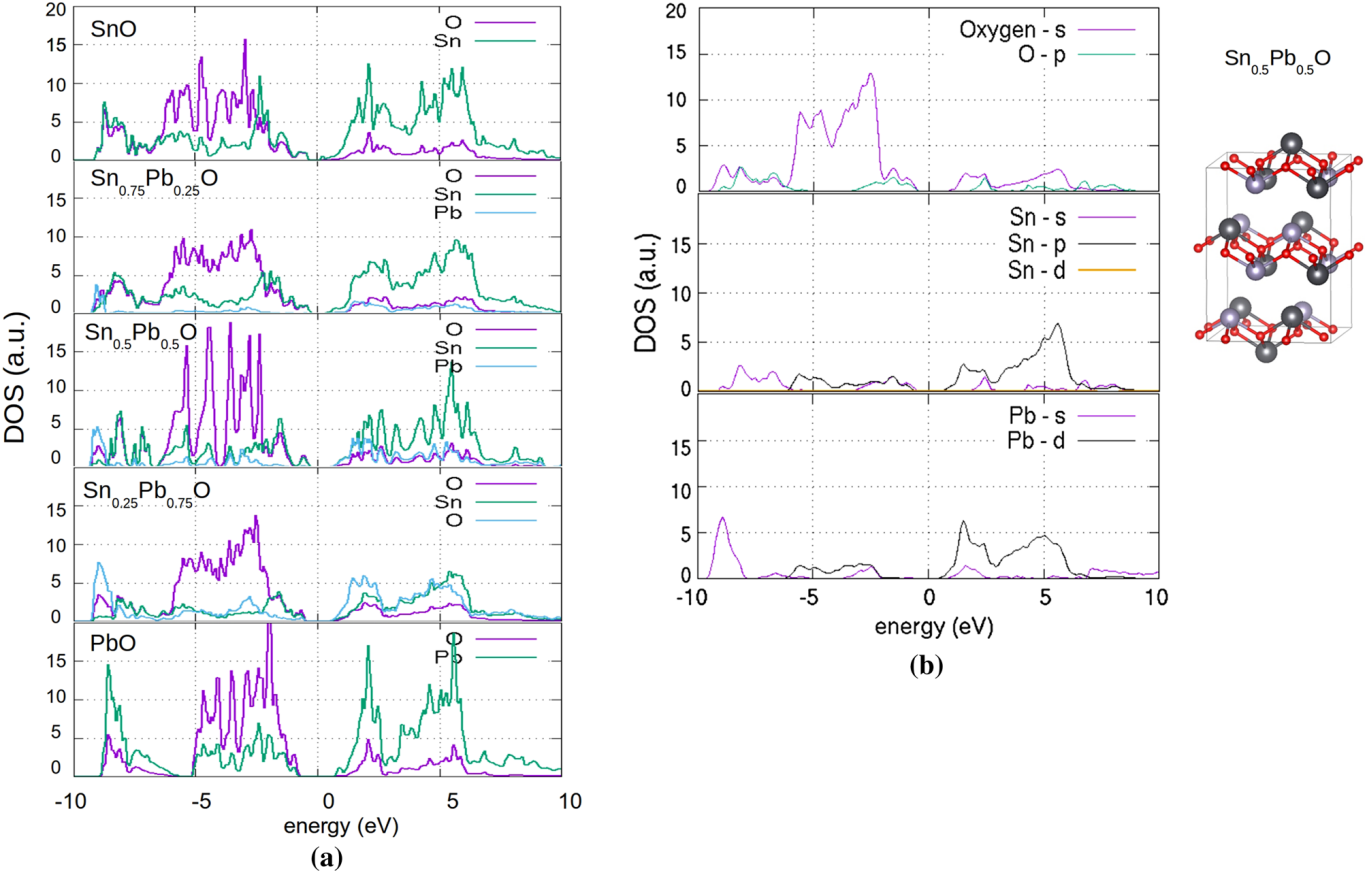
(**a**) Partial density of states versus Pb doping (**b**) projected DOS for Sn_0.5_Pb_0.5_O.

**Figure 5:**
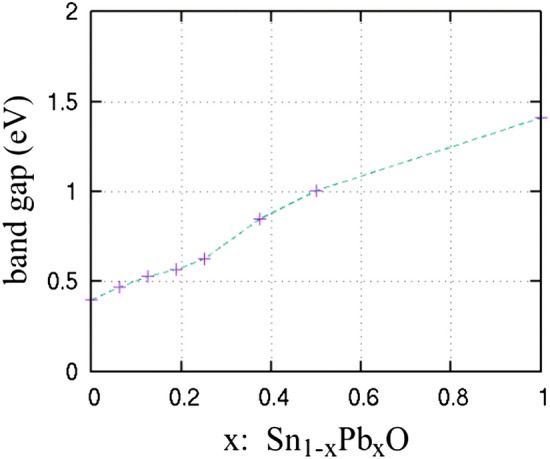
Calculated band gap with respect to the Pb content of Sn_1−x_Pb_x_O.

In Fig. 4a, the DOS plots of the Sn_x_Pb_1−x_O concentration for concentration x of 0, 0.25, 0.5, 0.75 and PbO (x = 1) are shown. In the case of Sn_0.5_Pb_0.5_O, the projected DOS is shown in Fig. 4 (b) along with the simulated structure (inset). The Pb s and p orbitals both contribute to the conduction band, along with the Sn p-orbitals. The valence band near the gap is mainly due to the oxygen with contributions of the p and s orbitals of Pb and Sn. At the region approximately 9 eV below the VBM, a peak is observed due to the bonding interaction between Pb 6p and O 2p orbitals, similar to those obtained in previous work^36^. The insertion of Pb in the SnO litharge lattice at Sn-substitutional positions increases monotonically the band gap. Additionally, the presence of isovalent Pb in the Sn_1−x_Pb_x_O does not induce defect states in the gap region of the alloys. The calculated band gap vs Pb (%) concentration is shown in Fig. 5. The energy band gap of Sn_1−x_Pb_x_O is found to increase almost linearly from 0.4 up to 1.4 eV as x is varied from 0 to 1. It is expected for the energy band gap of a ternary compound semiconductor A_x_B_1−x_C (0 < x < 1) to vary between the band gaps of the endpoint binary constituents AC and BC. The ability to tune the energy bandgap of this specific oxide i.e. Sn_1−x_Pb_x_O is important as it may be used as a p-type semiconductor that absorbs in the visible and consequently could be used in an all-oxide solar cell as an absorber layer. The absence of mid gap states is important. Mid gap states related to crystal imperfections in the energy gap would be detrimental to a solar cell absorber.

### Work function calculation

**Figure 6:**
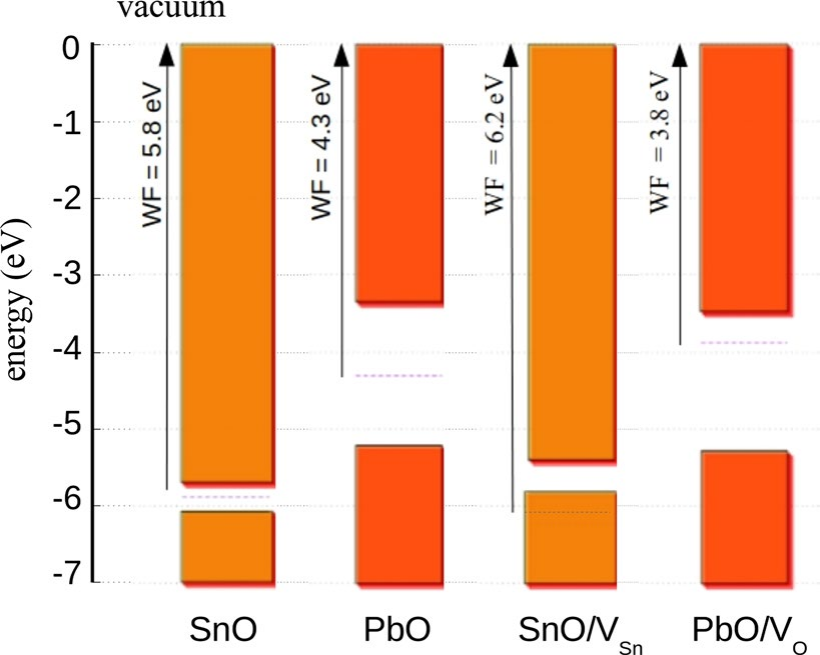
Band alignments of SnO, PbO, SnO with 2% V_Sn_ and PbO with 2% V_O_, with respect to the vacuum energy level.

We have performed DFT calculations in order to obtain the work function. For this scope, a SnO 2 × 2 × 6 supercell with a total of 96 atoms and a vacuum of 25 Å was used for the calculations and the average electrostatic potential along the z axis (001 direction) was calculated with the help of the c2x tool^37^. The work function, *W*, was then calculated as

$$W = V_{vac} - E_{F} ,$$

where *V*_*vac*_ is the vacuum potential and *E*_*F*_ the Fermi energy for the SnO and PbO structures. With this method, we have calculated the work function of SnO structures at 5.8 eV with a band gap of 0.5 eV and the work function of PbO structures at 4.3 eV with a band gap of 1.8 eV. The experimental value for the work function of SnO is 5.2 eV^10^. The work function of PbO has been measured between 4.8 and 5.5 eV, depending on growth conditions^38^. The energy band alignment of stoichiometric SnO and PbO is shown in Fig. 6. It is shown that PbO and SnO form a type—III heterojunction.

We examined the formation energy (E^f^) of the anion and cation defects for both SnO and PbO using the following equation, written here for the case of Sn vacancy:

$$E^{f} [V_{Sn} ] = E_{tot} [SnO_{{V_{Sn} }} ] - E_{tot} [SnO] + \mu_{Sn}$$

where E^f^[V_Sn_] is the formation energy of a Sn vacancy, E_tot_ [SnO_Vsn_] is the total energy of the SnO supercell containing the Sn vacancy, E_tot_[SnO] the total energy of the supercell and μSn the chemical potential of Sn. For SnO, the formation energy of a Sn vacancy is calculated at 1.66 eV, much lower than the formation energy of the O vacancy which is calculated at 4.33 eV. For PbO, the formation energies of the Pb vacancy is calculated at 3.99 eV, very close to that of an O vacancy which is 4.02 eV.

As mentioned earlier, SnO is often grown non stoichiometrically, with the existence of Sn vacancies that are reported to contribute to the p-type conductivity. Similarly, PbO is usually grown with O vacancies, responsible for its n-type conductivity^29^. Here we examine the effect of the defects most likely to be present on experiment on the electrical properties and work function, namely SnO with Sn vacancies and PbO with oxygen vacancies. We calculate the alignment of the energy levels for the perfect structures of SnO and PbO but also those that are related to experimental conditions. In the 226 supercell that we examine here, the Sn-poor SnO and Pb-rich PbO are calculated with a vacancy of an atomic concentration of 2.08%. The band alignment of the energy levels of the defected structures are shown in Fig. 6. The PbO and SnO structures show a type-III band alignment for both the perfect (PbO/SnO) and the defected (SnO + V_Sn_/PbO + V_O_) pairs. The calculated work function for each structure is shown in Table 2. Cation defects will increase the work function of SnO by 0.4 eV. The reduced (oxygen deficient) PbO shows a reduction of the work function by 0.5 eV.

**Table 2:** Calculated work function for perfect SnO, SnO with an atomic concentration of 2% Sn vacancies, PbO, PbO with an atomic concentration of 2% oxygen vacancies.

Structure	WF (eV)
SnO	5.8
SnO + V_Sn_	6.2
PbO	4.3
PbO + V_Ox_	3.8

The resulting p-n SnO/PbO heterojunction with a broken band gap alignment may be used in an-all oxide tandem solar cell. The lattice constants of SnO and PbO are very close indeed to each other as shown in Fig. 3 meaning that a high crystal quality heterojunction interface is feasible. This could be used as a p-n tunnel junction in tandem solar cells consisting of multiple layers of Sn_1−x_Pb_x_O with different energy band gaps modified by x similar to the Cu_2_O/In_2_O_3_ tunnel junction used in copper indium gallium diselenide tandem solar cells by Song et al.^39^*.* In addition to solar cells the properties of Sn_1−x_Pb_x_O ternary oxide and SnO/PbO heterojunction would also be very interesting for the efficient separation of photo excited electron–hole pairs during the photo-electro-chemical generation of oxygen and hydrogen i.e. water splitting.

The electronic properties examined here (band-gap, work function and band alignment) of few layer SnO and PbO structures are expected to show dependancy on the number of layers as in the example of Phosphorene^40^. Preleminary work shows that the band-gap of SnO is increased when decreasing the number of SnO layers, providing a very interesting pathway for the implementation of SnO 2D structures or SnO/PbO 2D heterostructures.

## Conclusion

The properties of Sn_1−x_Pb_x_O alloys are examined using first-principles calculations. Pb doping/alloying provides a pathway for tuning the band gap of SnO without introducing defect states in the region of the gap. In addition we find that for the PbO/SnO heterojunction is calculated to be a type-III heterojunction. When introducing defects in the system that are likely to be present in experiment, namely Sn vacancies in SnO and oxygen vacancies in PbO, the work functions of the non stoichiometric structures are altered. The work function of SnO is calculated to increase significantly, by 0.4 eV, when introducing 2% Sn vacancies whereas the PbO work function is expected to decrease by 0.5 eV for an atomic concentration of 2% oxygen vacancies. For this case, the type-III character of the heterojunction is retained, according to calculations. This broken gap band alignment is attractive for energy conversion in solar cells or photocatalysis and hydrogen generation.

## Methods

For our DFT calculations we utilize the CASTEP (CAmbridge Serial Total Energy Package)^41^ plane-wave/pseudopotential code. The exchange–correlation interactions are treated at the generalized gradient approximation (GGA) level with the density functional of Perdew, Burke and Ernzerhof (PBE)^42^ using ultrasoft pseudopotentials^43^. The cut-off energy of the plane wave basis is set at 580 eV. For the structural optimization calculations, the unit cell was optimized with a k-point grid of 7 × 7 × 7. To study the Pb doping for various dopant concentrations, a 3 × 3 × 3 supercell consisting of 108 atoms is used with a 2 × 2 × 2 Monkhorst–Pack (MP)^44^k-point sampling mesh for the optimization of the bulk structure. Additionally, a 2 × 2 × 2 supercell which consists of 32 atoms was employed for the higher dopant concentrations. For surface calculations, a 2 × 2 × 6 supercell of 96 atoms is employed with a vacuum of 25.4 A in the (001) direction and a k-mesh of 3 × 3 × 2 points. The energy tolerance for convergence is set at 10^–6^ eV and maximum ionic force tolerance at 0.05 eV A^−1^. Structural figures were generated using the VESTA visualization software^45^.
